# Recent advances in retroviruses via cryo-electron microscopy

**DOI:** 10.1186/s12977-018-0405-6

**Published:** 2018-02-23

**Authors:** Johnson Mak, Alex de Marco

**Affiliations:** 1 Institute for Glycomics, Griffith University Gold Coast, Southport, QLD Australia; 2 Department of Biochemistry and Molecular Biology, Monash University, Clayton, VIC Australia

**Keywords:** Cryo-electron microscopy, Cryo-electron tomography, Structural biology, Single particle, Subtomogram averaging

## Abstract

Cryo-electron microscopy has undergone a revolution in recent years and it has contributed significantly to a number of different areas in biological research. In this manuscript, we will describe some of the recent advancements in cryo-electron microscopy focussing on the advantages that this technique can bring rather than on the technology. We will then conclude discussing how the field of retrovirology has benefited from cryo-electron microscopy.

## Introduction

Biological systems are complex environments populated with millions of molecules that include structural proteins, enzymes, nucleic acids and lipids [1]. Many of these molecules interact with multiple partners in order to fulfil their role. Duration, stability and specificity of those interactions vary from one situation to the next [2] and understanding these intermolecular relationships can provide insights into their mechanisms of action. Data about a specific interaction can be used to develop computational models to predict how these molecules function [3–5], examples include the study of the interactome of YGL161G in Yeast [3]. In the case of pathogens, such structural knowledge can be used for the design of vaccines [6, 7] and novel therapeutics [8, 9]. Currently, the three major techniques commonly used for structural determination are X-ray crystallography, Nuclear Magnetic Resonance (NMR) and cryo-Electron Microscopy (cryoEM) [10]. All three of these approaches can be used to resolve the structure of a protein (or complexes) to atomic or near-atomic resolution. Through X-Ray crystallography and NMR, it has been possible to resolve the structure of isolated proteins or complexes in isolated states. Despite the significant information provided through these methods, the result can be prone to artefacts and poor interpretation. One reason for such drawback is that these approaches do not take into account the environment where the proteins or complexes normally exist. In this review, we will share the basic principles of cryoEM and highlight some of the recent advances, we will discuss advantages and potential pitfalls of this technique. In particular, we will provide examples of how cryoEM has revealed aspects of retrovirology that were previously unknown to us.

## Cryo-electron microscopy and related techniques

### The electron microscope

The term CryoEM covers a broad range of methods that share the common ground of imaging, through a transmission electron microscope (TEM), a radiation sensitive sample that is kept at cryogenic temperature [11]. A TEM provides a detailed map of the electron densities distributed across the inspected sample. Similarly, a light microscope provides a map of the optical density of a sample, but the resolution that can be achieved by these two techniques differs significantly due to the diffraction limit of the wavelength in use. Accordingly, using visible light, observable features are limited to ~ 200 nm resolution, while by using soft X-Rays the resolution limit is down to ~ 30 nm. To be noted that this limit depends on the current limitations in lens manufacturing. In contrast, electrons have a wavelength of ~ 2 pm at 300 keV and the lens system in these microscopes allow a resolution limit to the angstrom (Å) range. In this latter case, the resolution limit of the instrument is not limiting, while the structural variability within the samples together with their sensitivity to the electron beam will be the major limiting factors.

In TEMs, electrons are emitted by a source filament (that can be thermoionic or cold depending on the material and operation principle) and accelerated at voltages typically ranging between 60 and 300 keV. The electron beam is shaped and directed to the sample through an electromagnetic condenser lens system. While passing through the sample, each electron scatters differently depending on the local composition of the specimen (atomic cross-section) [12]. The scattered beam is then refocused through another set of electromagnetic lenses (objective lens) that will project a magnified image on a detector. Over the past few years, the development of new imaging detectors allowed for the capture of images with extremely low electron doses and short exposures. This is attributed to the ability to directly detect electrons (Direct Electron Detectors), having both a high quantum efficiency (up to 70% depending on energy and frequency) and fast readout speed (up to 400 frames/s). These advancements allow for collecting images with high signal-to-noise ratio at the higher frequencies (if compared to the film and CCD data). The poor signal-to-noise ratio is recognized as one of the major limiting factors in achieving high-resolution until 2012 [13, 14].

### The sample preparation

CryoEM analysis consists of imaging samples maintained at cryogenic temperatures (80–120 K). Cryogenic conditions limit the effects of radiation damage on biological samples [15] and provide a means for instantaneous fixation. In fact, if the freezing process is fast enough all the water in the sample will become vitreous and any activity (down to molecular level) will stop [15]. Vitreous ice is an amorphous solid form of water, which can withstand a high vacuum environment (such as the one found inside a TEM) without displaying significant sublimation. Vitreous ice also has the same electron transparency as liquid water making cryoEM ideal to inspect proteins. In fact, an electron accelerated at 300 keV (the most commonly used energy in high-resolution cryoEM) can travel through a region up to ~ 250 nm of water and statistically undergoing a single elastic scatter event (mean free path) [16].

Vitreous ice, however, is not a condition that is easily achieved. A rapid drop in temperature throughout the whole sample (from room temperature to cryogenic temperature) must be reached within ~ 1 ms. For a thin sample that is less than 10 µm, vitreous ice can be achieved through plunge freezing the sample in liquid ethane [15]. For thicker samples, the preferred method is High-Pressure Freezing (HPF). HPF consists of a rapid cooling of biological samples (even up to 200 µm in thickness) within ~ 2 ms, while the pressure is increased to 2000 bars to prevent the formation of crystalline ice [17].

The most common method of sample preparation (including purified proteins, protein complexes and viruses) requires deposition of a water-soluble substrate onto a TEM grid support that is covered with a holey carbon foil. Once the sample has been spread throughout the grid, excess liquid is removed by blotting in order to leave the thinnest possible film. This is then plunged into liquid ethane.

### Different samples require different approaches

Currently, cryoEM comprises of a multitude of applications from imaging of purified proteins to its application on intact entities. These include large macromolecular complexes, viruses, bacteria or even tissue sections. Depending on the sample and the question to be addressed, multiple potential workflows can be applied for cryoEM based structure determination. Currently, the preferred method to resolve the structure of purified proteins and protein complexes is Single Particle cryoEM (SP cryoEM). As previously mentioned, biological samples are extremely sensitive to high energy electrons, therefore images must be taken using a very low dose. Imaging at low dose lead to an extremely low signal-to-noise ratio, and since scattering is a stochastic event it can lead to incomplete sampling. Another limitation comes from the fact that molecules are three-dimensional objects, while electron micrographs are two-dimensional projections, which can, per se, only provide an incomplete description of the sample. The simplest way to overcome these problems consists of ‘averaging’ multiple copies of the same molecule which have been imaged from enough orientations to cover every view. This is to some extent analogous to what is done in X-ray crystallography, where the quality of the signal obtained in the diffractogram depends in part on the number of repeated unit cells present in the imaging area and the uniformity of the unit cells. By imaging multiple copies of the same molecule in random orientations, it is possible to obtain three-dimensional structural information of the complex in question. All projection images will be classified based on orientation and (if applicable) conformation. Once enough views have been identified and enough statistics are available for each of those, relevant images will be averaged and combined, through back-projection, to form a noise-free three-dimensional representation of the molecule under study. One of the major advantages of Single Particle cryoEM is that the crystallization step in X-ray crystallography may be bypassed. Furthermore, the structure can be determined while the molecule is in a state that is closer to physiological, rather than the solid form of a protein crystal that may or may not be biologically relevant. At the same time, the heterogeneity (of the structural folds in solution) resulting from the allowed flexibility can become a significant hurdle to providing high-resolution 3D reconstructions that are more readily achieved via X-ray crystallography [18]. An increasing number of algorithms are being developed to overcome this problem of heterogeneity and hopefully obtain a model describing all the states that a molecule can adopt in solution. As of today, this type of comprehensive description of state has been only possible for a restricted set of molecules [19].

Complexes that are organised following a defined high order symmetry (such as actin filaments, microtubules or enveloped viruses), can be extremely unlikely to crystallize. For those, a similar approach as described for single particle cryoEM can be used to determine the high-resolution structure of the complex. In this case, the high-order symmetry is advantageous since the symmetry helps to initiate the alignment by often providing strong low-resolution information. Furthermore, the symmetry provides multiple views of the same protein in each particle and the orientation between each monomer is defined by the symmetry. The two techniques that are mostly used to resolve the structure of the monomers for these type of symmetrical assemblies are helical and icosahedral reconstruction [18]. Owing to the fact that the sample flexibility is limited by the molecular packing and considering that each individual particle contains multiple views of the same object (inherent in the symmetry), both techniques demonstrated their potential leading to resolutions below 3Å even in times when cryoEM was a very niche field and automated microscopes and direct detectors were not available [20–26].

If one is interested in understanding the structural organization of complexes in their native biological context, these appear as the convolution of molecules that are located across multiple layers, the relative position of each molecule does not necessarily follow any high order symmetry nor any relationship. This type of arrangement is valid also for purified large macromolecular complexes such as viral capsids or vesicles that can be pleomorphic but still display local symmetry or order. Consequently, the determination of these structures cannot be achieved through single particle cryoEM. One potential way to overcome this conundrum is to produce a 3D representation of the sample by performing a tomographic acquisition. This technique is known as cryo-Electron Tomography (cryoET) and consists of imaging the same area from multiple different angles, which is then followed by a back-projection step. CryoET can be used to analyse the landscape of a cellular region or to resolve structures of proteins or protein complexes: the process required for these two different types of data collection changes significantly between these two applications.

Since tomographic acquisition is slow (40′—1 h/tomogram), regardless of whether the objective is to obtain a detailed description of the cellular landscape or determine the structure of the complex to high resolution, the magnification will be lowered to the minimum allowable level that would still resolve the structure of interest by maximising the field of view. For example, if the goal is to obtain landscape information at a resolution of ~ 3 to 5 nm, the electron dose will be maximised to the limit allowed by the sample (more than 100 e^−^/Å^2^) to provide the best possible contrast. Alternatively, should the goal be to obtain molecular structural information in details, the electron dose needs to be minimised to prevent damage (between 50 and 100 e^−^/Å^2^) during data acquisition, and the magnification can be increased in order to boost the signal to noise ratio at high-resolution. In fact, the camera response or efficiency in digitizing the image (Detector Quantum Efficiency or DQE) is higher in the middle and low-frequency range.

The ability to describe a sample in three dimensions through tomography depends on the ability to image it from different angles. A major limitation of cryoET is linked to the impossibility of tilting the sample up to 90°, as past 60°–70° the grid becomes too thick to image. Typically the resolution achievable from a single tomogram is comprised between 3 and 5 nm in XY (resolution here is limited by the maximum electron dose a sample can sustain) and lower in Z (depending on the completeness of the tilt range). This is the effect of the so-called missing wedge of information [27]. In order to eliminate the anisotropy derivations from the incomplete tilt range as well as boosting the signal (and therefore the resolution), one can apply an averaging for structural determination. This approach, called “subtomogram averaging”, is the equivalent of single particle analyses but performed on three-dimensional data instead of the projection data. Typically small subregions that are expected to contain the complex of interest are extracted from the tomograms (subtomograms), those are iteratively aligned and averaged. It has been shown that this approach is capable of providing near-atomic resolution, similarly to cryoEM Single Particle Analyses [28, 29].

### Hybrid techniques and correlative light and electron microscopy

Finding multiple copies of samples within an EM grid for imaging in nanometre resolution is like finding a needle in a haystack. A typical problem arising upon the inspection of a tomogram collected on a cell is that it appears as a constellation of densities, and only a small fraction of those densities can be unequivocally recognised through their shape and/or location from the sample grid for imaging. Over the past few years, the use of Correlative Light and Electron Microscopy (CLEM) has been extended toward cryopreserved samples, making it much easier to identify rare events (or protein complexes in this case) for structural determination [30–32]. Current developments in cryo-CLEM showed that it is possible to predict the position of a molecule in three dimensions with a precision within 200 nm [33,34], and it is possible (where needed) to thin a sample using cryo-focused ion beam (cryo-FIB) so that it would become compatible with cryoET [35, 36].

## Where did cryoEM help in understanding retroviral biology?

### Analyses of glycoproteins of SIV and HIV-1 enveloped viruses

For a long time, viral proteins have been among the most studied by structural biologists, not only for the high pathological relevance but also because a significant portion of the viruses studied over the years has a capsid which is organised following a strict icosahedral symmetry [37]. Glycoproteins in icosahedral viruses are generally distributed following the symmetry of the capsid. The structure in those cases can be obtained together with the capsid structure through cryoEM and icosahedral reconstruction [38]. With the implementation of sub-tomogram averaging, it is now possible to resolve the structure of viral glycoproteins located on the membrane of irregular enveloped viruses. Even without knowing the exact location of the target proteins, the typical workflow consists of the indiscriminate extraction of observed density located on the membrane surface followed by an iterative alignment. This methods works based on the idea that the most abundant feature will predominate in the final average, while all densities that do not match will be excluded based on correlation threshold when compared to the average. A few densities are manually picked based on the expected shape and size, which are then used to provide a reference model. The classification can be done by applying a threshold to the cross-correlation value calculated during the alignment with the reference or with the sum of the densities after alignment.

**Fig. 1:**
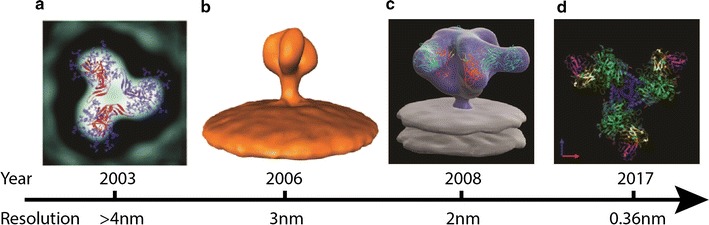
A chronological timeline of structural details of HIV envelope that has been unveiled via cryo-electron microscopy. Panel images** a**–**d** are modified from Zhu et al. [51], Zanetti et al. [106], Liu et al. [39] and Ozorowski et al. [54], respectively

Subtomogram averaging was used to successfully determine the structure of the gp120 trimer on the surface of SIV and HIV-1 virions at a resolution close to 2 nm [39, 40] (Fig. 1a). The analysis, in this case, allowed an understanding of the binding dynamics and conformational changes induced by the broadly neutralising antibodies (bNAb). Broadly neutralising antibodies have been a subject of intense research, and understanding how bNAbs can be elicited in human hosts would be vital for the development of HIV vaccine candidate. Generally speaking, there are five target sites for bNAbs against HIV envelope: the V2 site, the N332 supersite, the CD4 binding site, the gp120-41 interface, and the membrane proximal external region (MPER) (for review see Wibmer et al. [41]). The structural arrangement of the interaction between env and the antibody have been resolved through cryoEM [42–49].

Over the years there have been multiple attempts to resolve the structure of the envelope gp120 trimer for both SIV and HIV-1 [39,50–54] (Fig. 1b, d). In addition to an increase of the resolution limit as the technology improves, there are also visible differences between individual cryoEM structures that depend on the preparation of the envelope protein trimeric structure, such as a fully glycosylated cleaved version of the protein [44], a complex that involves binding with CD4 receptor, co-receptor or receptor mimic antibody [42, 45, 47, 49, 55–57]. The bindings between HIV envelope trimer with antibody, CD4 receptor or co-receptor generally leads to structural reorganization (review see [41, 49]). The combination of super-resolution microscopy and cryoEM has also suggested that the virion particle also undergoes a size expansion upon CD4 receptor engagement [58], which might be important to facilitate the subsequent biological process of HIV infection.

As previously discussed in [59], there have been evident differences in the processing methods used and the type of controls applied. This learning curve has helped to define a procedure to validate any structure that is computed through cryoEM or cryoET. Nonetheless, it is still important to be conscious that cryoEM is still a developing field, therefore there are still potential pitfalls that a novice can run into [60, 61], and incorporation of validation standards is currently considered a high priority for experts and developers in this field [62–64].

### Analyses of the retroviral capsid during viral assembly and maturation

It has long been known that retroviruses assemble an immature virion through the accumulation of multiple copies of its structural polyprotein Gag. This assembly process occurs underneath the plasma membrane of the host cells (with the exception of the spumaviruses and betaretroviruses where the assembly phase takes place in the cytoplasm). Immediately after budding, a set of proteolytic cleavages induce a dramatic change in the structural organisation. The macroscopic differences between mature and immature forms of the virus are striking enough that these are visible through conventional TEM analyses, but the real breakthrough in understanding the re-arrangement of Gag’s domains throughout this process came from the application of cryoET [65]. In fact, the inherent pleomorphism present across all retroviruses made it impossible to obtain a detailed model of the virions through other methods such as X-ray crystallography.

**Fig. 2:**
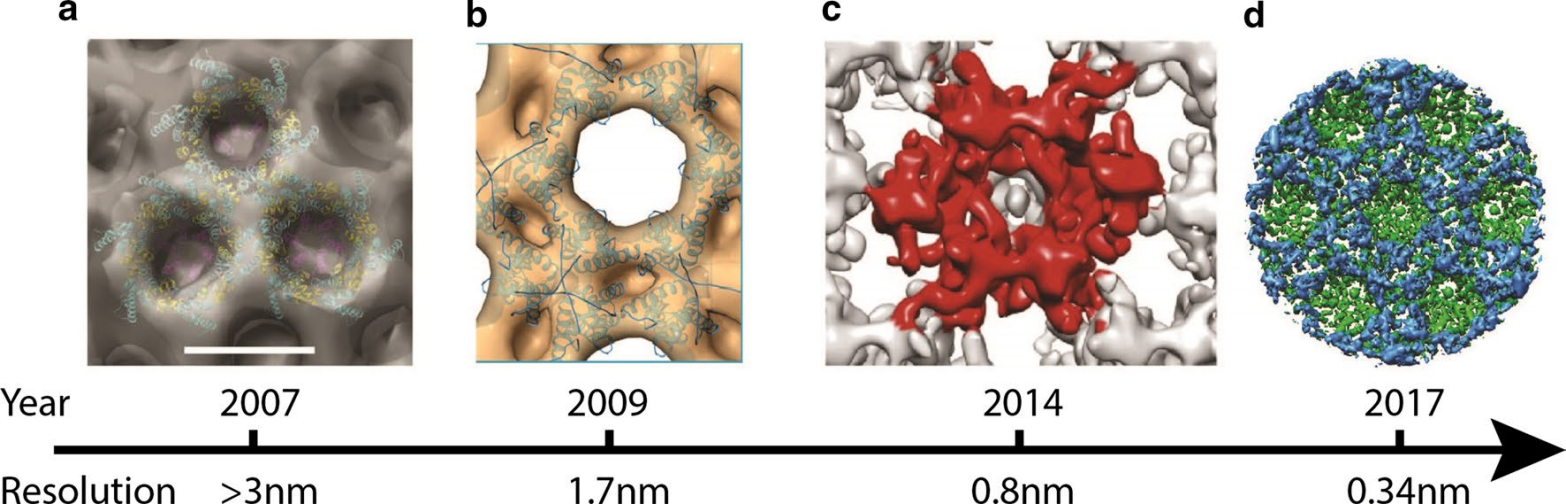
A chronological timeline of structural details of HIV capsid that have been unveiled via cryo-electron microscopy. Panel images ** a**–**c** are modified from Wright et al. [66], Briggs et al. [67], Bharat et al. [107], while the** d** refers to Turonova et al. [29] and was downloaded from EMDB.org

The early studies on immature virions were conducted on lentiviruses, specifically on HIV-1. It was shown that Gag assembles underneath the membrane and this layer can adopt multiple curvatures and some patches appeared disordered [66]. Later it was shown that the Gag layer is organised to form a hexagonal lattice, with an 8 nm spacing (Fig. 2), and that the changes in curvature are accommodated through the introduction of imperfections in the lattice [67]. Thanks to the recent improvements in both hardware and image processing tools, over the past years the resolution at which the organisation of the immature Gag lattice was resolved went from multiple nanometers to near atomic [28] (Fig. 2). The immature Gag lattice has been studied and its structure has also been resolved for alpha and betaretroviruses (respectively for Rous Sarcoma Virus—RSV—[68] and Mason-Pfizer Monkey Virus—M-PMV—[69]). For all these studies, the advancements in cryoET have been critical, in fact, early-low resolution–reconstructions performed on all those viruses suggested a high level of conservation throughout CA [70]. Recently it has been possible to reconstruct the lattice of immature virions for all these three viruses at significantly higher resolutions [68, 71], those structures showed that the arrangement of CA in the C-terminal domain (CTD) is well conserved across the family, while the N-terminal domain (NTD) appear to differ significantly. For example, the NTD in alpha retroviruses (RSV) have large contacts to ensure a tight interaction between neighbouring hexamers along the lattice. In addition, it appears that RSV stabilises the lattice through the interactions of the p10 domain [72].

It is well known that a common step for retroviruses is the maturation. The virion buds out from the infected cell in immature and non-infectious form. Upon the activation of the viral protease, the polyprotein Gag is cleaved in multiple positions, and these induce a dramatic rearrangement of the virion core, leading to the formation of an infectious particle [73]. HIV-1, because of its pathological importance, is the virus whose maturation process has been studied the most. Macroscopically the changes include the rearrangement of the lattice from an incomplete sphere to a fullerene cone and the increase of the spacing in the CA lattice from 8 to 10 nm [73]. The maturation process in HIV-1 occurs through 5 sequential proteolytic cleavages whose order and rate have been measured [74] and their roles in the maturation have been analysed through infectivity and morphological studies [75, 76]. Although the cleavage order in nature is probably not as strict as in vitro, and cleavages on different molecules might happen on different sites at the same time, the data available as of today [74] suggest that the cleavage between SP1 and NC is the first proteolytic processing event to occur, and it leads to the detachment of the capsid-based lattice from the NC proteins (therefore the RNA genomes) [76]. The second step is the detachment of the C-terminus p6 domain from the NC domains, this is required for a proper reorganisation of the RNA genome and it is needed for the infectivity of HIV [75]. (3) The third proteolytic cleavage event occurs between MA and CA, and this event induces the most dramatic change in the virion. Consequently, the CA lattice is reorganised completely, and it no longer displays the 8 nm hexameric arrangement upon MA-CA cleavage [76–78]. The fourth proteolytic cleavage event separates NC from SP2, which leads to genome condensation, although this cleavage appears not to be strictly required neither for infectivity nor proper genome condensation [75]. The final cleavage event is critical for the infectivity and to allow re-assembly of the core in the mature form [76, 79]. The last cleavage event, which has been intensely studied, is the site where novel anti-retroviral maturation inhibitor Bevirimat acts, which prevents the formation of an infectious virion. Through cryoET studies, it is now appreciated that Bevirimat suppresses HIV maturation by stabilising the immature CA lattice intermediate to prevent further maturation by denying viral protease access to the cleavage site [28, 77]. Thanks to the use of cryoET, it has been possible to identify the effects of allosteric integrase inhibitors (ALLINIs), which impair the maturation displaying electron-dense aggregates located next to a malformed mature capsid [80].

In general, the structure of immature, mature and “maturing” virions, could not have been determined without the application of cryoET because retroviruses are pleomorphic. If every virion is different, then classical structural biology techniques, which are based on averaging multiple identical objects are inapplicable. As of today, the only way to obtain a good description of viruses that lack symmetry—and more generally any object composed of multiple layers that are oriented independently from each other—is to perform a tomographic acquisition. An example of this need comes from the evidence that in multiple cases the mature core of HIV-1 is not always a fullerene cone. The models obtained previously showed that the mature core in HIV-1 follows the geometry of the fullerene, based on information obtained mostly from projection images [81–84] and fitting the structure of hexamer and pentamer CA assembly that had been crystallised [82, 85]. The first sub-nanometre 3D reconstruction of a mature like lattice on a tubular assembly showed that a three-helix bundle is critical for the lattice assembly, as it provides a strong set of hydrophobic interactions [86]. Recently the mature CA lattice has been solved at 8 Å directly in the virus [87]. To highlight the importance of conducting structural studies with minimal purifications and if possible directly in situ, the comparison between the existing crystal structures [82, 85] and the EM structure showed a most evident crystallisation artefact around the structure of the CA pentamer [87]. While it is known that the most common form of the mature CA core is a fullerene cone, it has been frequently reported that other conformations such as cylindrical cores, spherical as well as non-closed or incomplete shells, are possible [84, 87, 88]. Currently, there are two models proposed for the mature core formation: a de novo assembly which was proposed through computational models [85] and is currently supported by the available high-resolution reconstructions obtained from multiple mature virions and [87, 89]. An alternative model proposes the phase transition as non-diffusional but as the result of a gradual conversion which would roll on itself to form a mature core [90]. Frank et al. also produced for the first time evidence of a possible trimeric arrangement of the MA layer [90].

### The HIV-1 intasome

Another aspect of HIV biology that has benefited a great deal from cryo-EM is our understanding of the integration process. Upon infection, retroviruses integrate their genome into the host cell chromosomes. This integration process results in a permanent presence of the retroviral genome within the host cell [91]. Retroviral integration is carried out by the viral integrase, which oligomerises and complexes with the viral DNA to form the stable synaptic complex (SSC). The stable synaptic complex (also known as intasome) will subsequently transport into the nucleus and facilitate the insertion of the viral genome into the host [91, 92].

Structural studies of the intasome have been attempted for many years because of its relevance as a potential antiretroviral target, including the refinement of integrase inhibitors. The main challenge in studying intasome is the tendency of the intasomes to aggregate, making the crystallisation process extremely complicated. As of today, the intasome of RSV [93] and PFV [94] have been successfully crystalised, however, thanks to the simpler requirements for cryoEM in structural determination, the structures of four additional intasomes have been resolved. The structure of the PFV intasome/nucleosome complex has been resolved at 0.78 nm resolution [95] providing the bases for understanding the mechanism behind the nucleosome capture by the retroviral integration machinery. In addition, the structure of the intasome from two more genera have now been resolved, including the Betaretroviruses (on MMTV) [96] and Lentiviruses (on HIV-1) [97]. Lately, the structure of the lentiviral intasome nucleoprotein complex (obtained from the Maedi-visna virus (MVV)) was obtained at 0.49 nm resolution [98]. This structure has been proposed as a platform for drug design of HIV-1 IN inhibitors.

## Correlative microscopy: a help to find the needle in the haystack

As mentioned in the introduction, despite the great resolution that cryoEM can deliver, biological samples are extremely complex and only a subset of the molecules imaged in a cryoEM micrograph can be unequivocally identified. Moreover, most of the biological samples are pleomorphic, meaning that it is impossible to predict with high certainty the location of a molecule or a complex. A major consequence of this incomplete understanding of the sample is that locating objects and/or events that are rare and smaller than 1 µm can be an extremely challenging and time-consuming activity. Moreover, there will be numerous cases where the complex might not be unequivocally identifiable. Here the aid provided by the combination of light microscopy with electron microscopy is invaluable and typically is referred as correlative microscopy. A number of different combination of correlative microscopy approaches have been described based on the objectives of the research questions [31, 35, 99].

This technique has been successfully used to image rare events linked to the retroviral replication cycle such as viral budding and entry. Early studies using correlative light and scanning electron microscopy showed that it was possible to identify with micrometric precision the location of a bud on an infected cell [100]. Other cases displayed a simplified approach where the focus was on the identification of cells that had been infected, in order to speed up the screening [101] and allow the identification of locations presenting viral buds for further structural analyses. Another rare and isolated event in the replication cycle is the viral entry. Here free virions have been identified through live cell imaging, their movements have been followed and the cells after plunge freezing have been inspected first through cryo-fluorescence microscopy and then through cryoEM [34, 99].

## Final notes

The objective of this review was to provide an overview of the emerging capabilities available for structural studies through cryo-electron microscopy that can be of aid in the field of retrovirology. The impact that Cryo-EM and related techniques can be seen throughout all fields related to cellular biology. While electron microscopy was temporarily “out of fashion” as a research tool near the end of the 20th century; the recent technological advances, together with the wider spread of cryoEM have already shown how the structural determination of large protein complexes at near atomic resolution is possible for an increasing number of molecules. Cryo-electron microscopy, more specifically, single particle cryoEM was recognised as ‘Method of the year in 2015’ by Nature Methods [102] and the subject of 2017 Nobel Prize in Chemistry. With the continuous advancement of the field, it is now possible to perform cryoEM on larger assemblies, such as entire pleomorphic virus particles. The recent advances in image processing for cryoET showed that it is possible to achieve near atomic resolution on isolated pleomorphic virions [28, 29], in the future, we can expect a routine combination of cryoET on cellular samples through the use cryo-Focused Ion Beam Milling. As this combination has already allowed the resolution of structures within the cell [36, 103–105] it is reasonable to expect an improvement of resolution in the coming future. It is an exciting time for cryo-electron microscopy and virology in general.
